# Comparison of the Effects of 0.05% Difluprednate Ophthalmic Solution and 1% Prednisolone Acetate Ophthalmic Solution on Intraocular Inflammation Following Lens Surgery in Healthy Rabbits

**DOI:** 10.3390/vetsci13101087

**Published:** 2026-10-14

**Authors:** Hiroaki Okui, Shoma Takishita, Naoyuki Fuke, Yasuyuki Kaneko, Satoshi Sekiguchi, Kiyokazu Naganobu

**Affiliations:** 1 Graduate School of Medicine and Veterinary Medicine, University of Miyazaki, Miyazaki-shi 889-1601, Miyazaki, Japan; ophvethiro@gmail.com; 2 Kurume Dog and Cat Hospital, 1949-12 Kokubu-machi, Kurume 839-0863, Fukuoka, Japan; 3 Department of Veterinary Science, Faculty of Agriculture, University of Miyazaki, 1-1 Gakuen-Kibanadai-Nishi, Miyazaki-shi 889-2192, Miyazaki, Japan; takishita-s9887@pref.fukuoka.lg.jp (S.T.);; 4 Laboratory of Veterinary Pathology, Department of Veterinary Science, Faculty of Agriculture, University of Miyazaki, 1-1 Gakuen-Kibanadai-Nishi, Miyazaki-shi 889-2192, Miyazaki, Japan; 5 Veterinary Teaching Hospital, Faculty of Agriculture, University of Miyazaki, 1-1 Gakuen-Kibanadai-Nishi, Miyazaki-shi 889-2192, Miyazaki, Japan; yasuyuki-kaneko@miyazaki-u.ac.jp; 6 Center for Animal Disease Control, University of Miyazaki, 1-1 Gakuen-Kibanadai-Nishi, Miyazaki-shi 889-2192, Miyazaki, Japan

**Keywords:** difluprednate, prednisolone acetate, rabbit, phacoemulsification

## Abstract

Prednisolone acetate (PA) ophthalmic solution is used to treat post-cataract surgery uveitis. In humans, studies comparing difluprednate (DFBA) and PA ophthalmic solutions have reported the usefulness of DFBA. Herein, we compared the effects of PA and DFBA on uveitis following phacoemulsification in rabbits by evaluating anterior chamber flare values using a laser flare meter, anterior chamber flare grades using a slit-lamp microscope, posterior capsule opacification (PCO) formation, and histopathological findings. Anterior chamber flare values were highest on the first postoperative day in both groups, decreasing thereafter. Flare values were significantly lower in the DFBA group than in the PA group only on postoperative day 7; no significant between-group differences were observed at other measurement points. Flare grades were highest on the day after surgery in both groups and subsequently decreased, with no significant between-group differences. There were no significant differences in PCO area or the number of inflammatory cells in the iris, ciliary body, and choroid. These findings suggest that the suppressive effect of DFBA on uveitis following phacoemulsification in healthy rabbits is comparable to that of PA. Future studies should investigate the effects of DFBA ophthalmic solution in cataract surgery in rabbits and other animal species.

## Simple Summary

In recent years, cataract surgery is performed more frequently in veterinary medicine, and 1% prednisolone acetate (PA) ophthalmic solution is widely used to suppress postoperative inflammation. Conversely, in human medicine, 0.05% difluprednate (DFBA) has recently come into use. Unlike PA, DFBA does not require shaking before administration, offering greater convenience. This study compared the anti-inflammatory effects of these agents following lens surgery (phacoemulsification) in rabbits. Anterior chamber flare values, a measure of intraocular inflammation determined by a laser flare mater, was highest on the day after surgery in both groups, decreasing thereafter. Flare values on postoperative day 7 were significantly lower in the DFBA group than in the PA group. There were no differences in the numbers of inflammatory cells in the iris, ciliary body, and choroid on histopathological examination. These findings suggest that the suppressive effects of DFBA on uveitis following phacoemulsification in healthy rabbits is comparable to that of PA. Future studies should investigate the effects of DFBA ophthalmic solution in cataract surgery in rabbits and other animal species.

## 1. Introduction

Cataracts are one of the leading causes of vision loss in animals, and lens surgery is accordingly widely performed to restore visual function [1]. Currently, phacoemulsification is the standard surgical method, and surgical success rates have improved thanks to advances in surgical techniques and equipment; for example, one report indicated that vision was maintained in 94.8% of cases following diabetic cataract surgery [2]. However, intraocular surgeries, such as cataract surgery, are accompanied by surgical trauma that can induce uveitis [1]. Postoperative uveitis can cause corneal edema, anterior chamber fibrin deposition, posterior synechiae, glaucoma, retinal detachment, and posterior capsule opacification (PCO), all of which can significantly worsen visual prognosis; therefore, appropriate postoperative anti-inflammatory therapy is essential [1,3]. Corticosteroid eye drops are often used to suppress postoperative inflammation, with 1% prednisolone acetate (PA) being widely recommended for animals [1,3]. PA is also widely used for anti-inflammatory treatment following human cataract surgery. Recently, the use of 0.05% difluprednate (DFBA) eye drops after human cataract surgery and comparisons with PA have been reported [4,5,6,7,8]. Multiple randomized controlled trials and meta-analyses have shown that the efficacy of DFBA is equal to or greater than that of PA in treating inflammation following human cataract surgery [5,6,7,8]. Furthermore, because PA is poorly soluble in water, it is formulated as a suspension; reports indicate that insufficient shaking prior to use or improper storage conditions can cause the administered drug concentration to widely vary from 7.0% to 231.5% of the labeled concentration [4]. In contrast, 0.05% DFBA ophthalmic solution is a derivative of prednisolone formulated as an emulsion; as such, it does not require shaking, consistently delivers a uniform dose, and exhibits excellent penetration into the cornea and intraocular tissues [4,9].

In animals, a study using a paracentesis-induced uveitis model in healthy Beagle dogs reported that both DFBA and PA were effective at suppressing anterior chamber flare, an indicator of uveitis [8]. Another report noted that aqueous humor protein and PGE2 concentrations were lower in eyes treated with DFBA compared to those treated using betamethasone [10]. Although similar paracentesis models have been reported in rabbits, to the best of our knowledge, no studies have yet compared the anti-inflammatory effects of DFBA and PA following lens surgery in animals. Rabbits are commonly used in research involving intraocular lenses after phacoemulsification, and cataract surgery is also often performed on pet rabbits, during which PA eye drops are typically administered postoperatively [11,12]. Therefore, this study aimed to perform lens surgery on healthy rabbits and compare the effects of DFBA and PA ophthalmic solutions on postoperative uveitis.

## 2. Materials and Methods

This study was conducted with the approval of the Experimental Animal Committee of Miyazaki University. Ten male pigmented rabbits (Dutch strain, body weight 1.4 ± 0.2 kg, 8 weeks old) were used in the experiment. All animals were housed individually in a room set at 20–24 °C with a 12 h light/dark cycle. The rabbits were determined to be healthy based on physical and blood examinations. Intraocular pressure was measured (Tono-Pen VET, Medtronic Solan, Jacksonville, FL, USA) under sedation using an intramuscular injection of 0.3 mg/kg medetomidine hydrochloride (Medetomin Injection, Meiji Seika Pharma, Tokyo, Japan). Subsequently, a combination ophthalmic solution comprising a tropicamide and phenylephrine hydrochloride (Mydrin^®^-P Ophthalmic Solution, Santen Pharmaceutical Co., Ltd., Osaka, Japan) was instilled into both eyes to induce mydriasis. Slit-lamp examination (SL-130, Carl Zeiss Meditec, Jena, Germany) and fundus examination using a binocular indirect ophthalmoscope were subsequently performed to confirm the absence of any abnormalities. Anterior chamber flare evaluation was performed by illuminating the anterior chamber using the smallest light beam available on the slit lamp. Slit-lamp images were video-recorded, and still images were extracted and saved from the videos. Additionally, baseline anterior chamber flare values were measured using a laser flare meter (FM-500, Kowa, Tokyo, Japan) prior to the experiment. The results of these slit-lamp examinations and anterior chamber flare evaluations were used as the preoperative data.

This study utilized DFBA ophthalmic emulsion (Sterop^®^, Senju Pharmaceutical Co., Ltd., Osaka, Japan) and PA ophthalmic suspension (Prednisolone Acetate Ophthalmic Suspension USP 1%, Alcon, Fort Worth, TX, USA). The rabbits were randomly divided into either the DFBA (*n* = 5) or PA group (*n* = 5) by S.T. using a coin flip, and phacoemulsification was performed only on the right eye of each rabbit (as described below). The surgery was performed by H.O., the slit-lamp examination and laser flare meter examination were performed by K.N., and the histopathological examination was performed by N.F. Both H.O. and K.N. were not informed which eye drops were used until all observations other than histopathological examinations were completed. N.F. was not informed which eye drops were used until histopathological examinations were completed. Eye drops were administered by S.T.; however, because the eye drop bottles differed between DFBA and PA, S.T. could not be blinded to the eye drops used.

In the DFBA group, one drop each of DFBA and 0.3% ofloxacin ophthalmic solution (Tarivid Ophthalmic Solution 0.3%, Santen Pharmaceutical Co., Ltd., Osaka, Japan) were instilled into the right eye of the rabbits. Similarly, one drop each of PA and 0.3% ofloxacin ophthalmic solution were instilled into the right eye of rabbits in the PA group. PA was thoroughly shaken and agitated before use. The two medications were administered approximately 5 min apart. Following instillation, the animals were sedated using an intramuscular injection of 0.3 mg/kg medetomidine. A combination an ophthalmic solution of tropicamide and phenylephrine hydrochloride was subsequently instilled into the right eye of each animal to induce mydriasis, and an intravenous catheter was inserted into the auricular vein. Approximately 30 min following medetomidine administration, anesthesia was induced by intravenous injection of 1 mg/kg alfaxalone (Alfaxan, Meiji Seika Pharma, Tokyo, Japan). A supraglottic airway device for rabbits (v-gel^®^ Rabbit, Acoma Medical Industry, Tokyo, Japan) was placed, and general anesthesia was maintained with isoflurane (Mylan, Tokyo, Japan) and oxygen. Following the initiation of isoflurane, 5 mg/kg of enrofloxacin (Baytril, Bayer Yakuhin, Tokyo, Japan) was injected intramuscularly. With the rabbit restrained in left lateral recumbency, the hair surrounding the eyelids and periocular area was clipped, and the eyelids and ocular surface were disinfected with an iodine-containing ophthalmic irrigating and disinfecting solution (PA·IODO Ophthalmic and Eye washing Solution, Nihon Tenganyaku Kenkyusho, Nagoya, Japan). The ocular surface was washed with the iodine-containing ophthalmic irrigating and disinfecting solution diluted 50-fold with physiological saline and then rinsed with physiological saline. After draping, the ocular surface was disinfected again with the 50-fold diluted iodine-containing ophthalmic irrigating and disinfecting solution and washed with physiological saline. Using a crescent knife and a slit knife, a two-plane corneal incision was made near the limbus. Following the injection of a viscoelastic material (Suvenil Joint Injection, Chugai Pharmaceutical Co., Ltd., Tokyo, Japan) into the anterior chamber, a continuous curvilinear capsulorhexis of the anterior lens capsule was performed using anterior capsule forceps. The lens was then removed, preserving the capsule using a phacoemulsification system (SOVEREIGN^®^ Compact Phacoemulsification System, Abbott, Tokyo, Japan). The intraocular irrigating solution used comprised a mixture of 500 mL of BSS Plus intraocular irrigating solution 0.0184% (BSS Plus 500, Alcon Japan, Tokyo, Japan), 0.5 mg of adrenaline injection (Bosmin^®^ Injection 1 mg, Daiichi Sankyo, Tokyo, Japan), and 1000 units of heparin (Heparin Na Injection 5000 units/5 mL, Mochida Pharmaceutical, Tokyo, Japan). The phacoemulsification system was set at a bottle height of 50 cm, ultrasound power of 60%, and an aspiration flow rate of 20 mL/min. Following completion of lens processing, the corneal incision was closed with simple interrupted sutures using 10-0 nylon (Mani Nylon, Mani, Tochigi, Japan). Upon completion of surgery, 0.02 mg/kg of buprenorphine (Lepetan^®^ injection, Otsuka, Tokyo, Japan) was administered subcutaneously. Atipamezole hydrochloride (0.15 mg/kg; Antisedan^®^, Nippon Zenyaku Kogyo Co., Ltd., Fukushima, Japan) was then injected subcutaneously to facilitate recovery from anesthesia.

Postoperatively, starting on the day of surgery, the DFBA group received DFBA and 0.3% ofloxacin ophthalmic solution four times a day in the right eye. The PA group received PA and 0.3% ofloxacin ophthalmic solution following the same regimen. An interval of at least 5 min was maintained between the instillations of the two drugs. These treatments were continued until postoperative day 28.

Slit-lamp examinations and laser flare meter examinations were performed on postoperative days 1, 4, 7, 14, 21, and 28. All examinations were conducted in the same darkened room. Fifteen minutes prior to these examinations, a combination ophthalmic solution of tropicamide and phenylephrine hydrochloride was instilled into both eyes to induce mydriasis. The rabbits were then sedated with an intramuscular injection of 0.3 mg/kg medetomidine hydrochloride.

For laser flare measurements, the average of five measurements was used, provided that the background difference before and after each measurement was within 20% [13]. However, for one rabbit in the DFBA group on postoperative day 4, only three measurements had a background difference within 20%; therefore, their average was used. Furthermore, based on the slit-lamp examination, the severity of anterior chamber flare and the presence or absence of fibrin in the anterior chamber were evaluated. Anterior chamber flare was graded from saved slit-lamp images using a modified classification adapted from a veterinary text [14]. The grades were defined as follows: 1+ for faint flare (barely detectable), 2+ for moderate flare (details of the iris and lens clearly visible), 3+ for marked flare (details of the iris and lens obscured), and 4+ for the presence of fibrin.

After the final observation on postoperative day 28, the rabbits were euthanized via an intravenous overdose of pentobarbital (Somnopentyl, Kyoritsu Seiyaku, Tokyo, Japan), and the globes were collected for histopathological examination. The globes were enucleated immediately following euthanasia and sectioned in half slightly anterior to the equator on the corneal side for evaluation of PCO; the lens capsule was then observed from the vitreous side using a slit lamp and video-recorded. Still lamp images were subsequently extracted from the video, the formation line of the PCO was manually traced on a computer, and the percentage of the PCO formation area was calculated using ImageJ (Version 1.51, https://imagej.net/ij/), defining the total area of the lens capsule as 100%.

Following PCO observation, the aforementioned globes were fixed in 10% neutral-buffered formalin and sectioned to preserve the continuity of the cornea, ciliary body, and iris. The tissues were embedded in paraffin, 3 µm sections were prepared, and hematoxylin and eosin (HE) staining was performed. Four contiguous sections containing the iris, ciliary body, and choroid were examined per eye.

Histopathological evaluation was performed using a Nikon Eclipse Ni microscope (Nikon, Tokyo, Japan) equipped with a 40× objective lens and a 10× eyepiece. Images were acquired at 400× total magnification using the Nikon DS-L4 imaging system. For each section, inflammatory cells were counted in 10 microscopic fields at 400× magnification. The area of each microscopic field was 0.237 mm^2^, resulting in a total evaluated area of 2.37 mm^2^ per section. Counting was initiated from the beginning of each anatomical structure (ciliary body, choroid, and iris), and microscopic fields were selected within the observable tissue area according to the same criteria for all samples. The terminal portions of these structures were excluded because they could not be consistently identified or observed in all specimens. Thus, the same anatomical criteria, magnification, and counting procedure were applied to all samples.

Inflammatory cells were identified morphologically based on their characteristic nuclear and cellular features. Mononuclear cells and neutrophils were observed and counted as inflammatory cells and were distinguished from surrounding resident tissue cells based on their morphological characteristics. The inflammatory cell count for each eye was defined as the mean count across the four contiguous sections.

Additionally, inflammatory findings, such as posterior synechiae, were evaluated.

### Statistical Analysis

Data are expressed as the median (range). Operating and phacoemulsification times were compared between the two groups using the Mann–Whitney U test. Changes in laser flare meter values and flare grades within groups over time were compared using the Steele method. The Mann–Whitney U test was further applied to compare the flare values measured with a laser flare meter and flare grade between the two groups at the same time point and to compare the number of postoperative days until fibrin disappeared and the proportion of PCO area between groups. The Wilcoxon signed-rank exact test was used to compare the number of inflammatory cells determined by histopathological examination between the operated and non-operated eyes in the same animal. The number of inflammatory cells on histopathological examination in the operated eye was compared between groups using the Mann–Whitney U test. Statistical analysis was performed using EZR (Version 1.52) [6]; EZR is available on CRAN (The Comprehensive R Archive Network) as the “RcmdrPlugin.EZR” package. Statistical significance was set at *p* < 0.05.

## 3. Results

The surgical times were 20 (16–22) minutes for the DFBA group and 17 (15–20) minutes for the PA group, while the respective phacoemulsification time were 138 (98–206) seconds and 124 (108–158) seconds; there were no significant differences between the two groups in either parameter.

The results from the laser flare meter examination are presented in Table 1. Preoperative flare values were 7.62 (5.26–9.38) pc/ms for the operated eyes in the DFBA group and 7.88 (6.64–8.48) pc/ms for the operated eyes in the PA group, with no significant differences observed between the two groups. Furthermore, the flare values for the non-operated eyes were 7.24 (4.20–7.72) pc/ms in the DFBA group and 7.74 (3.96–8.98) pc/ms in the PA group, with no significant differences observed between the non-operated and operated eyes in either group. In the operated eyes, flare values were highest in both groups on postoperative day 1 (DFBA group: 148.84 [52.42–206.14] pc/ms; PA group: 133.78 [74.66–261.18] pc/ms); however, no significant difference was observed between the two groups. Thereafter, flare values gradually decreased. On postoperative day 7, the flare value was significantly lower in the DFBA group (21.48 [16.08–29.52] pc/ms) than that in the PA group (48.68 [24.60–169.66] pc/ms). On postoperative day 14, although there was no significant difference, the DFBA group (8.46 [7.02–10.84] pc/ms) tended to show lower values compared to those of the PA group (13.74 [8.80–74.90] pc/ms, *p* = 0.0553). No significant differences were observed between the two groups at other observation points. Flare values in the non-operated eyes exhibited no significant changes throughout the observation period in both groups.

The flare grade based on slit-lamp photographs reached its maximum value on postoperative day 1 in both groups and subsequently decreased (Table 2). Throughout the entire observation period, no significant differences were noted in flare grades between the two groups at the same examination points.

On the day after surgery, fibrin formation was observed in the anterior chamber of all animals in both the DFBA and PA groups. The median number of postoperative days at which anterior chamber fibrin was last observed was 1 (1–4) days in the DFBA group and 4 (1–7) days in the PA group, with no significant difference observed between the two groups.

**Figure 1:**
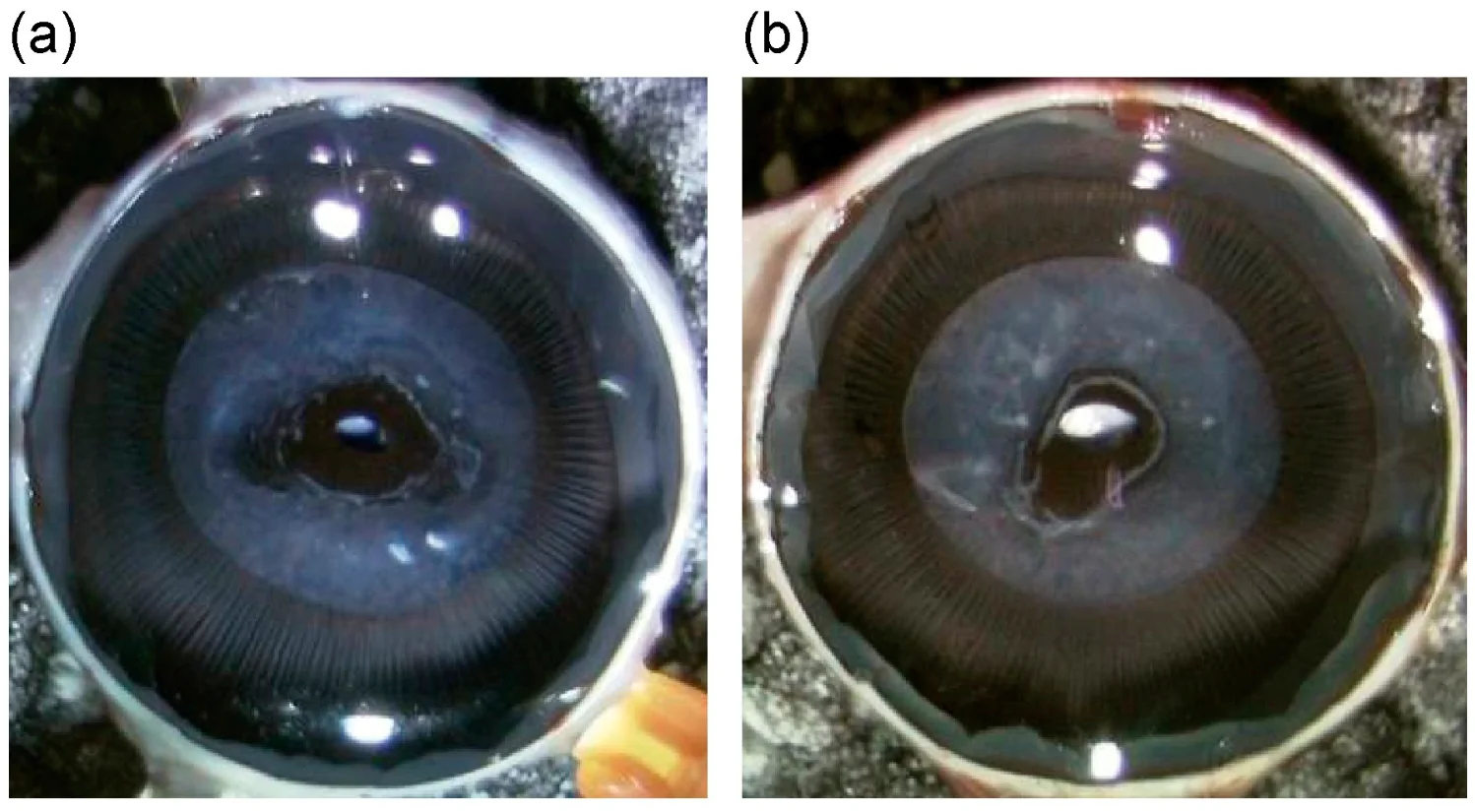
Examples of posterior capsule opacification observed 28 days after surgery. The excised eyeballs were cut in half near the equator, and the posterior capsule opacification was observed from the vitreous side using a slit lamp. (**a**) Rabbit treated with DFBA; (**b**) rabbit treated with PA.

The formation of a Soemmering’s ring, a type of PCO, was observed in all cases in both the DFBA and PA groups (Figure 1). The percentage of the PCO formation area relative to the total lens capsule area was 86.8% (78.6–89.6%) in the DFBA group and 84.3% (78.8–88.5%) in the PA group, demonstrating no significant difference between the two groups.

**Figure 2:**
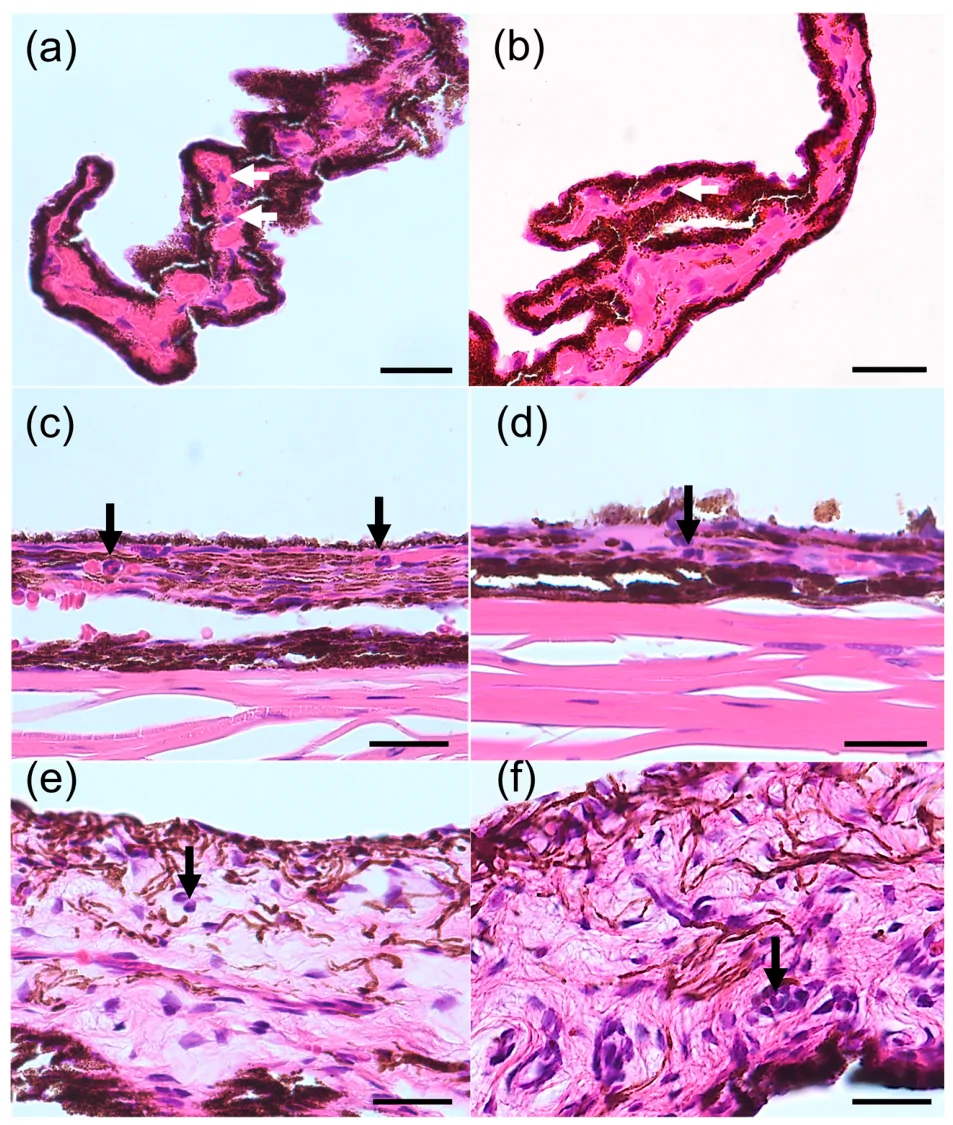
Representative histopathological images of the ciliary body, choroid, and iris. (**a**,**c**,**e**) Rabbit treated with DFBA; (**b**,**d**,**f**) rabbit treated with PA. Hematoxylin and eosin staining. Arrows indicate inflammatory cells. Bar = 30 μm.

In the histopathological examinations, the number of inflammatory cells was 2.5 (0.5–6.3) in the non-operated eyes of DFBA-treated rabbits and 5.0 (0.8–5.8) in their operated eyes, with no significant difference between the two (Figure 2, Table 3). Furthermore, the value was 3.8 (1.0–4.8) in the non-operated eyes of PA-treated rabbits and 3.8 (2.0–5.0) in their operated eyes, with no significant difference between the two. Moreover, there was no significant difference in the number of inflammatory cells between the operated eyes of DFBA- and PA-treated rabbits. Histopathological examination revealed no findings suggestive of inflammation, such as posterior synechiae.

## 4. Discussion

Anterior chamber flare is a useful indicator for evaluating uveitis. The aqueous humor is produced from blood plasma in the ciliary body; however, due to a mechanism known as the blood–aqueous barrier, large molecules such as proteins normally do not enter the anterior chamber. However, under uveitis conditions, this mechanism breaks down, resulting in an increase in the protein concentration of the aqueous humor. When a slit beam is directed into the anterior chamber under these conditions, the light scatters as it strikes the protein molecules, causing the Tyndall effect. This phenomenon is referred to as anterior chamber flare; the greater the disruption of the blood–aqueous barrier, the stronger the anterior chamber flare. Flare can be evaluated subjectively by slit-lamp examination or quantitatively using a laser flare meter or fluorophotometry.

In the present study, anterior chamber flare was evaluated both quantitatively using a laser flare meter and subjectively from slit-lamp images obtained during examination. Both of these measured flare values increased significantly on postoperative day 1 in both groups, decreasing thereafter. Neither group showed any significant changes in flare values or grades in the non-operated eyes. Therefore, these increases were considered to be related to the breakdown of the blood–aqueous barrier associated with inflammation caused by surgical trauma. On postoperative day 7, laser flare meter values were significantly lower in the DFBA group than those in the PA group. In contrast, no significant difference between the two groups was observed in the slit-lamp flare grades. This discrepancy was thought to arise because laser flare meter values are continuous numerical data, while flare grades represent a stepwise evaluation, potentially leading to different flare values being assigned the same grade. Additionally, fibrin formation in the anterior chamber was observed in all cases. When the blood–aqueous barrier is severely compromised, fibrin may precipitate into the anterior chamber. In the present study, there was no statistically significant difference in the number of postoperative days at which anterior chamber fibrin was last observed between the DFBA and PA groups. Thus, regarding the suppression of anterior chamber flare and fibrin precipitation, DFBA was considered to have an effect equivalent to that of PA.

Histopathological examination on postoperative day 28 revealed no significant difference in the number of inflammatory cells between the operated and non-operated eyes in either group. In dogs, uveitis can persist for a long period following cataract surgery [15]. Therefore, it is possible that both DFBA and PA had successfully suppressed uveitis by postoperative day 28 in the present study. Furthermore, no significant difference in the number of inflammatory cells was observed between the operated eyes of the two groups. In the evaluated tissue specimens of both the DFBA and PA groups, findings suggestive of inflammation such as posterior synechiae were not observed. Combined with the effects on anterior chamber flare described above, these findings indicate that DFBA may have an effect equal to that of PA against uveitis following lens surgery in rabbits.

In humans, several studies have reported that DFBA has an anti-inflammatory effect following cataract surgery equivalent to or greater than that of PA. One study comparing DFBA and PA administered six times daily following cataract surgery evaluated anterior chamber flare and cells, finding no significant differences between the groups during the 4-week postoperative evaluation period, indicating that DFBA was as effective as PA for the treatment of postoperative inflammation [16]. Other studies have likewise reported equivalent anti-inflammatory effects for DFBA and PA after human cataract surgery [13,17]. In contrast, KhalafAllash et al. concluded in their systematic review and meta-analysis that DFBA was significantly more effective than PA at reducing anterior chamber flare and cells 2 weeks after human cataract surgery.

There are few reports comparing DFBA and PA in animals. In one experimental study on healthy dogs, the effects of DFBA and PA administered either twice daily or four times daily on aqueocentesis-induced anterior uveitis were investigated using automated fluorophotometry. This study concluded that DFBA and PA, each administered twice or four times daily, showed a similar efficacy at suppressing the breakdown of the blood-aqueous barrier caused by aqueocentesis [18]. The results of the present study were consistent with those reported in studies comparing DFBA and PA following human cataract surgery and in canine models of aqueocentesis-induced anterior uveitis.

Formation of a Soemmering’s ring, a form of PCO, was observed in both groups. Following phacoemulsification, residual lens epithelial cells proliferate and differentiate into new cortical fibers, resulting in PCO formation [19]. A ring-shaped formation of cortical fibers between the posterior capsule and the residual anterior capsule is known as a Soemmering’s ring, which can also occur after phacoemulsification in rabbits [20]. One study on rabbits reported that diclofenac sodium and bromfenac sodium eye drops prevented the progression of PCO [21]. Furthermore, after cataract surgery and intraocular lens implantation in dogs, eyes implanted with a celecoxib-impregnated intraocular lens showed a significantly lower incidence of PCO than eyes treated with bromfenac eye drops at 4 weeks postoperatively, while eyes treated with bromfenac eye drops had a significantly lower incidence of PCO compared to eyes treated with PA eye drops at 56 weeks postoperatively [22]. PCO is known to involve the epithelial–mesenchymal transition of lens epithelial cells mediated by cyclooxygenase-2 [19]. Therefore, considering that differences among anti-inflammatory drugs may influence PCO formation, we compared the PCO areas. However, because no significant difference was observed between the two groups, DFBA and PA appeared to have similar effects on PCO formation through postoperative day 28.

These findings indicate that, in rabbits, DFBA may suppress uveitis following phacoemulsification as effectively as PA. Therefore, DFBA may be a potential anti-inflammatory treatment following cataract surgery in rabbits. Unlike PA, which is formulated as a suspension and must be thoroughly shaken before use to maintain uniformity, DFBA is formulated as an emulsion and therefore does not require agitation administration, representing a practical advantage. Consistent with the present findings, studies of human cataract surgery and canine aqueocentesis-induced anterior uveitis have additionally reported that DFBA is equivalent to or more effective than PA [5,6,7,8].

However, the present study had certain limitations. The small sample size limits the generalizability of the findings, and further research with a larger cohort is warranted. Histopathological examinations were performed only on postoperative day 28. Therefore, the histological changes during the early postoperative period, when flare values or flare grades were high, remain unclear. Additionally, the corneal effects, intraocular pressure, and systemic effects of DFBA and PA were not evaluated. The use of corticosteroid eye drops is known to cause corneal diseases [23] and systemic abnormalities such as iatrogenic Cushing’s syndrome. Therefore, potential adverse effects must be carefully evaluated when using DFBA in animals. Intraocular pressure may rise following cataract surgery. Since intraocular pressure was not evaluated in this study, it should be assessed in future studies.

## 5. Conclusions

In the present study, the effects of PA and DFBA eye drops on uveitis following phacoemulsification in rabbits were compared by evaluating anterior chamber flare values using a laser flare meter, anterior chamber flare grades using a slit-lamp microscope, PCO formation, and postoperative histopathological findings. As a result, no significant difference was detected between DFBA and PA in the suppressive effect on postsurgical uveitis following lens surgery under the conditions of the present study in rabbits. Future studies should investigate the effects of DFBA ophthalmic solution in cataract surgery in rabbit and other animal species.

## Figures and Tables

**Table 1:** Postoperative changes in flare value (photon count/msec), as measured with a laser flare meter.

Group	Preoperative	Days After Surgery					
		1	4	7	14	21	28
Operated eye							
DFBA	7.62 (5.26–9.38)	148.84 * (52.42–206.14)	33.32 * (16.22–38.66)	21.48 *† (16.08–29.52)	8.46 (7.02–10.84)	8.72 (5.44–25.60)	35.08 * (9.48–58.02)
PA	7.88 (6.64–8.48)	133.78 * (74.66–261.18)	41.36 * (19.54–101.62)	48.68 * (24.60–169.66)	13.74 * (8.80–74.90)	11.84 (4.98–24.28)	26.32 (6.12–61.58)
Non-operated eye							
DFBA	7.24 (4.20–7.72)	7.02 (6.10–8.84)	7.12 (5.12–9.10)	7.26 (4.46–8.36)	6.82 (5.36–8.24)	5.36 (3.94–8.66)	7.76 (4.26–15.60)
PA	7.74 (3.96–8.98)	7.1 (5.60–8.32)	4.88 (3.92–6.64)	5.58 (3.76–8.82)	7.1 (5.16–8.12)	5.84 (3.22–7.28)	8.28 (6.08–8.84)

Data are expressed as the median (range) from five animals per group, except for the PA group on postoperative day 28, which includes data from four animals. DFBA: difluprednate, PA: prednisolone acetate. * *p* < 0.05 vs. preoperative. † *p* < 0.05 vs. PA.

**Table 2:** Changes in flare grade observed on slit-lamp examination.

Group	Preoperative	Days After Surgery					
		1	4	7	14	21	28
Operated eye							
DFBA	0 (0–0)	4 (4–4) *	1 (1–4) *	1 (0–1)	0 (0–1)	0 (0–2)	1 (1–2) *
PA	0 (0–0)	4 (4–4) *	4 (1–4) *	2 (1–4) *	1 (0–1)	0 (0–2)	1 (0–2)
Non-operated eye							
DFBA	0 (0–0)	0 (0–0)	0 (0–0)	0 (0–0)	0 (0–0)	0 (0–0)	0 (0–0)
PA	0 (0–0)	0 (0–0)	0 (0–0)	0 (0–0)	0 (0–0)	0 (0–0)	0 (0–0)

Data are expressed as the median (range) from five animals per group. DFBA: difluprednate, PA: prednisolone acetate. * *p* < 0.05 vs. preoperative.

**Table 3:** Number of inflammatory cells in the ciliary body, choroid, and iris.

Group	Non-Operated Eye	Operated Eye
DFBA	2.5 (0.5–6.3)	5.0 (0.8–5.8)
PA	3.8 (1.0–4.8)	3.8 (2.0–5.0)

Data are expressed as the median (range). The inflammatory cell count for each eye was defined as the mean count across the four contiguous sections. DFBA: difluprednate, PA: prednisolone acetate.

## Data Availability

The raw data supporting the conclusions of this article will be made available by the authors on request.

## Abbreviations

The following abbreviations are used in this manuscript:

PA	Prednisolone acetate
DFBA	Difluprednate
PCO	Posterior capsule opacification
